# Muci specific IGE to modify myeloid derived cells of the tumor microenvironment

**DOI:** 10.1186/2051-1426-1-S1-P245

**Published:** 2013-11-07

**Authors:** J Mollick, M Madiyalakan, C Nicodemus

**Affiliations:** 1 Div of Oncology, Stanford University, Stanford, CA, USA; 2 Quest PharmaTech, Edmonton, AB, Canada; 3 AIT Strategies, Franconia, NH, USA

## 

We have used antibody (MAb) specific to hypoglycosylated tumor associated MUC1 as a strategy to induce tumor specific immunity. Xenotypic IgG1κ has been previously shown to induce T cell immunity targeting MUC1 in patients. We now describe MUC1 specific IgE to reprogram myeloid cells in the tumor microenvironment and modulate tumor specific immunity.

To test if these MAb mediate rejection of mouse breast cancer cells transfected with human MUC1 (4T-1/hMUC1), we injected MAb peritumorally for 5 days after sc tumor inoculation. These experiments were performed in mice with mFcεRI deleted, and transgenic for the counterpart human receptor (hFcεRI). We observed modest differences in the tumor growth rates after injection of hMUC1 IgE compared to a non-specific hIgE antibody. Analysis of the treated tumors showed solid sheets of tumor cells without the presence of effector cells of the allergic response.

Since MUC1 is a "neutral" antigen, and does not transmit a growth signal, we speculated that the antibodies modest activity was due to the absence of bone marrow derived cells in the microenvironment. Furthermore, the mice generated mouse anti-human IgE (MAHA) antibodies after two weeks. Accordingly, we engineered a 4T-1 tumor cell line that produced mouse IgE specific for hMUC1 (mIgE hMUC1). We also engineered other 4T-1 cells to produce hMUC1 and cytokines to draw myeloid cells into the tumor microenvironment. Cell mixing experiments were performed to bring together mIgE, the antigen (hMUC1) and the cytokines (IL-5 and MCP-1). These experiments, performed in KO/Tg animals, showed complete rejection of tumors in which MAb, antigen, and myeloid effector cells are brought together. If one of these elements were left out, the tumors grew progressively. We also observed that the presence of the two cytokines (IL-5, MCP-1) caused 4T-1 cells to metastasize early after injection.

To test if complete tumor rejection could only be observed in our KO/Tg mice, we repeated the cell mixing experiments in wild type Balb/c mice. In mixing experiments that brought together the antibody (anti-hMUC1 mIgE), the antigen (hMUC1) and the cytokines (IL-5/MCP-1), we observed slow growth of the tumors in Balb/c mice, suggesting that the expanded spectrum of expression of the hFcεRI was crucial for the complete elimination of the tumors from these KO/Tg mice. The presence of the IgE antibody reversed the "early metastases" phenotype conferred by the cytokines. Tumor antigen specific IgE is a promising approach for reprogramming myeloid cells in the tumor microenvironment, leading to elimination of tumors, and reversal of the cytokine-conferred metastatic phenotype. These antibodies may also be useful for augmenting host tumor immunity.

